# Cultural Adaptation of an Innovative Reproductive Health Assessment Tool for Dementia Research in Africa: Insights from the Fember‐Africa Study

**DOI:** 10.1002/alz70858_104321

**Published:** 2025-12-25

**Authors:** Alice Moraa Ondieki, Anne Nyambura Njogu, Cynthia Isabel Smith, Rachel W Maina, Edna N Bosire, Karen Blackmon, Harrison Kaleli, Sarah Gregory, Jasmit Shah, Elena Tsoy, Jennifer S. Yokoyama, Sheila Waa, Anusha Yasoda‐Mohan, Violet Okech, Udunna Anazodo, Michelle M Mielke, Tamlyn J Watermeyer, Chinedu Udeh‐Momoh

**Affiliations:** ^1^ Brain and Mind Institute, Aga Khan University, Nairobi, NAIROBI, Kenya; ^2^ Brain and Mind Institute, Aga Khan University, Nairobi, Kenya; ^3^ Brain and Mind Institute, Aga Khan University, Nairobi, Nairobi, Kenya; ^4^ Brain and Mins Institute, Aga Khan University, Nairobi, Kenya; ^5^ Edinburgh Dementia Prevention, University of Edinburgh, Edinburgh, United Kingdom; ^6^ Aga Khan University, Nairobi, Kenya; ^7^ University of California San Francisco, San Francisco, CA, USA; ^8^ Global Brain Health Institute (GBHI), University of California San Francisco (UCSF); & Trinity College Dublin, San Francisco, CA, USA; ^9^ Global Brain Health Institute/ Trinity College Dublin, Dublin, ireland, Ireland; ^10^ kenyatta National Hospital, Nairobi, Kenya; ^11^ Montreal Neurological Institute, McGill University, Montreal, QC, Canada; ^12^ Division of Public Health Sciences, Wake Forest University, School of Medicine, Winston‐Salem, NC, USA; ^13^ Edinburgh Dementia Prevention, Edinburgh, United Kingdom; ^14^ Female Brain Health and Endocrine Research (FemBER) Consortium, Newcastle, Edinburgh, London, United Kingdom; ^15^ Global Brain Health Institute, University of California, San Francisco, NC, USA; ^16^ Wake Forest University, School of Medicine, Winston‐Salem, NC, USA

## Abstract

**Background:**

Dementia research accounts for only 0.1% of all research in Africa, making it the lowest among all low‐ and middle‐income country (LMIC) regions. The development and adaptation of biological and psychosocial measures in ethnically and culturally diverse populations remain limited but are essential for culturally informed research. This is particularly critical for examining sex‐ and gender‐based vulnerabilities to Alzheimer's disease and related dementias (ADRD), including factors such as reproductive health and fertility.

**Method:**

We conducted a thorough review of our clinical and health questionnaires for cultural relevance and sensitivity through a series (*n* = 3) of focus groups discussions. These focus groups included a diverse range of participants, such as expert clinical and academic stakeholders, local community members, health promoters, community leaders, and representatives, ensuring a well‐rounded and inclusive approach.

**Result:**

Certain questions about sexual behavior, sexually transmitted diseases, biological and adopted children, and fertility were deemed culturally inappropriate and required rephrasing for sensitivity. To build rapport, these questions were strategically placed after less sensitive topics. Additionally, gaps were identified, including missing questions on traditional fertility practices (e.g., herbal remedies), male puberty characteristics, and partner support during and after childbirth. Addressing these gaps by incorporating local beliefs and traditions will enable a more holistic understanding of reproductive health behaviors. Furthermore, translations overlooked subtle linguistic nuances, highlighting the need for more detailed explanations or alternative concepts in Swahili to ensure clarity and accuracy.

**Conclusion:**

The Fember‐Africa study aims to bridge a critical gap in understanding sex‐ and gender‐specific differences in Africa, shedding light on the disproportionately higher prevalence of dementia among women of African ancestry. Through the culturally sensitive adaptation of reproductive health assessment tool, the study seeks to generate valuable insights that can inform the prevention and management of Alzheimer's disease and related dementias (ADRD) in this underrepresented population.

